# Heritable *Epichloë* symbiosis shapes fungal but not bacterial communities of plant leaves

**DOI:** 10.1038/s41598-019-41603-5

**Published:** 2019-03-27

**Authors:** Riitta Nissinen, Marjo Helander, Manoj Kumar, Kari Saikkonen

**Affiliations:** 1 Department of Biological and Environmental Science, P.O. Box 35, 40014 University of Jyväskylä, Jyväskylä, Finland; 2 Department of Biology, 20014 University of Turku, Turku, Finland; 3 Biodiversity Unit, 20014 University of Turku, Turku, Finland

## Abstract

Keystone microbial species have driven eco-evolutionary processes since the origin of life. However, due to our inability to detect the majority of microbiota, members of diverse microbial communities of fungi, bacteria and viruses have largely been ignored as keystone species in past literature. Here we tested whether heritable *Epichloë* species of pooidae grasses modulate microbiota of their shared host plant.

## Introduction

Microbe-plant interactions have existed since the origin of plants, and microbes have been demonstrated to contribute to all major aspects of plant life, including regulation of metabolism and growth, nutrient acquisition and resistance to biotic and abiotic stressors^1,2^. They can thus potentially modulate all ecosystems because food chains rely on primary producers converting energy from the sun to the consumers.

Traditionally, research on plant-microbe interactions has focused on one or very limited number of microbes or microbial groups. However, recent developments in molecular methodology have dramatically extended our understanding of plant microbiota, and demonstrated that the interactions between the plant and its associated microbiota, as well as microbe-microbe interactions within microbiome, impact strongly plant phenotypes and that plant microbiota are at least partially heritable^3^. Thus, the plant and its associated microbiome should be regarded as a co-evolving ecosystem, holobiont^1^. Here we propose that the most topical steps forward in biology are to untangle how selection treats plants and their symbiotic microbes individually or in concert as a holobiont, and to unravel how individual keystone microbial species drive the rest of the microbiota associated with plants, and thus potentially determine the plant performance and ecosystem functions relying on the plant^4,5^.

Here we test the hypothesis that individual keystone microbial species can modulate plant microbiomes using fungal endophytes of the genus *Epichloë* as a model. They commonly colonize cool-climate grasses^6^. *Epichloë*-grass symbiosis has been intensively studied since the 1970s when alkaloids produced by them were discovered to cause animal disorders^7^. Since then, *Epichloë* species have been shown to be able to modulate virtually all plant-plant, plant-herbivore and plant-pathogen interactions^8,9^ and thereby shape community structures of grassland ecosystems. Thus, they can be regarded as keystone species. Although the ecological importance of *Epichloë* species is demonstrated and widely recognized, their interactions with plant-associated microbes other than plant pathogens have largely been ignored (but see^10,11^). Here we propose that the *Epichloë* species, while growing systemically throughout the above ground parts of the host grass including the developing seeds, are likely to interact with a broad spectrum of taxonomically diverse microbial species occupying the host endosphere. They can e.g. compete, interact chemically and modulate plant quality to other microbes^12^. This calls for comprehensive community-level studies to test whether *Epichloë* species affect the rest of the grass microbiota.

To empirically approach the hypothesis that *Epichloë* species act as keystone microbial species modulating plant microbiomes we analyzed *Epichloë coenophiala* mediated changes in the bacterial and fungal endophytic communities of tall fescue grasses. As systemic *Epichloë* colonization has been reported to strongly modulate plant chemistry and physiology^6,13–15^, we expected that the bacterial and fungal community structures would be shaped by the *Epichloë* colonization status of the shared plant host.

## Materials and Methods

### Plant material and experimental setup

In 2003, a stock population of *Epichloë* colonized (E+) and endophyte-free (E−) tall fescue (*Schedonorus phoenix* (Scop. Holub.) [=*Lolium arundinaceum* (Schreb.) Darbysh. = *Schedonorus arundinaceus* (Schreb.) Dumort]) seeds collected from eight naturally occurring populations from Åland Island (Finland) was established in the Turku University Botanical Garden (60°26 N, 22°10.4E). A mixture of mother plant seeds, either E+ or E− tall fescue, were used in the experiment. Because the fungus is maternally inherited, the endophyte status of grasses (E+ or E−) used in the experiments was confirmed by microscopic examination of 1–3 seeds of each mother plant. The hyphae of the endophyte are visible by light microscopy among the embryonic cells of the plant^16^ after the seeds have been soaked overnight in solution containing water, ethanol and NaOH. Ten 1 m × 1.2 m tall fescue plots were established, 2.5 m apart from each other, in the Botanical Garden on the 25th of May 2016. Approximately 300 g of E− or 380 g of E+ seed mixture were sown to the plots. The higher amount of seed material was sown in E+ plots because this mixture had more non-seed material (seed envelopes etc.) compared to E− mixture. Every other plot was sown with E+ seeds, and every other with E− seeds. The seeded plots were covered with white mesh, which was removed after two weeks of germination. The plots were watered when needed, but not fertilized, as the soil in the plots (decomposed slurry) was expected to contain high amount of nutrients.

### Sample collection and processing

In August 15, 2016 three samples of *Schedonorus phoenix* fully grown, undamaged green leaves were collected from each plot, resulting in a total of 15 E+ and 15 E− samples. Samples were transported on ice to the laboratory and processed within 8 hours after harvesting. The leaves were thoroughly washed with water and surface sterilized by immersing in 70% ethanol for 1 minute, followed by 3% sodium hypochlorite for 3 min and sterile double distilled water (3 × 90 s). 300 mg of leaf blade from mid-leaf, including both vascular and leaf mesophyll tissue were excised with a sterile blade and homogenized in liquid nitrogen with a mortar and pestle. 80–100 mg of each sample was used for subsequent DNA analysis. Prior to DNA isolation, samples were homogenized with a sterilized mortar and pestle in liquid nitrogen. An Invisorb Spin Plant Mini Kit (STRATEC Biomedical AG, Germany) was used for community DNA isolation according to the manufacturer’s protocol.

16S rRNA gene was amplified following the protocol described in^17^ using primers 799 f/1492r^18^ and M13-1062f/1390r^19^ in a nested approach. The nested primers targeting the V6-V8 regions of 16S rRNA gene enable elimination of plant chloroplast 16S rRNA gene amplicons as well as separation of endophyte amplicons from plant mitochondrial amplicons by size fractionation (799f–1492r)^18^ and produce an amplicon with high phylogenetic coverage and optimal size for IonTorrent sequencing (1062f–1390r)^20^. Primer 1062 f was tagged with a M13 sequence to enable sample barcoding as described in^19^. Both reactions had 1 μl of sample DNA, 1x PCR buffer, 1 mg/ml of BSA, 0.2 mM dNTP’s, 0.3 μM of each primer and 1250 U/ml GoTaq DNA Polymerase (Promega, WI, USA) in a 30 μl reaction volume. 30 ng of DNA was used in the first PCR, and 1 μl of 1:10 diluted amplicons from the first PCR were used as a template for the second run. PCR reactions were performed as follows: 3 min denaturation at 95 °C followed by 25 cycles of denaturing, annealing, and extension at 95 °C for 45 s, 54 °C for 45 s, and 72 °C for 1 min, respectively. Final extension was carried out at 72 °C for 5 min.

Sequencing libraries were prepared by running a third PCR to attach the M-13-barcode system^19^. Amplicons from the second PCR were diluted 1:5 and re-amplified using barcode sequence-M13 system as forward primers and 1390r-P1 with adaptor A as a reverse primer. PCR mix and conditions were similar as described above, with an exception of using 8 cycles for amplification. Amplified libraries were purified using Agencourt AMPure XP PCR purification system (Beckman Coulter, CA, USA). Purified samples were quantified with Tape Station 2200 (Agilent, CA, USA) and were pooled based on an equivalent quantity of endophyte amplicon per sample. The pooled samples were size fractionated (size selection range of 350–550 bp) using Pippin Prep (Sage Science, MA, USA) 2% Agarose gel cassette (Marker B) following the manufacturer’s protocol. Size fractioned libraries were sequenced using an Ion 314 chip kit V2 BC on Ion Torrent PGM (Life Technologies, CA, USA) in the Biocenter Oulu, Finland.

Fungal internal transcribed spacer (ITS) gene libraries were amplified using fITS7 and ITS4 primer sets^21^. The 30 µl reaction mixture contained 1 μl (30 ng) of sample DNA, 1x PCR buffer, 1 mg/ml of BSA, 0.2 mM dNTP’s, 0.3 μM of each primer and 1250 U/ml GoTaq DNA Polymerase (Promega, WI, USA) in a 30 μl reaction volume. The amplification procedure consisted of 5 mins denaturation at 95 °C followed by 35 cycles of denaturing, annealing, and extension at 95 °C for 30 s, 55 °C for 30 secs and 72 °C for 1 min, respectively. Final extension was carried out at 72 °C for 7 mins. The sequencing libraries were prepared by attaching a M-13 barcode system to the amplicons as described above and in^20^. Amplicons from the first PCR were diluted 1:10 and re-amplified using a barcode attached M13 system as a forward primer and ITS4-P1 with adaptor A as a reverse primer. PCR mix and conditions were similar as described above, with the exception of only using 8 cycles for amplification. Amplified products were purified with an Agencourt AMPure XP PCR purification system (Beckman Coulter, CA, USA) and quantified using a Qubit dsDNA HS assay kit and Qubit fluorometer (Invitrogen, MA, USA). The quantified samples from each sample were pooled equimolarly and were sequenced using an Ion 314 chip kit V2 BC on Ion Torrent PGM (Life Technologies, CA, USA) in the Biocenter Oulu, Finland.

### Bioinformatics and statistical analyses

The sequence reads were processed using a CLC Genomics Workbench 11.0 with a Microbial Genomics Module (Qiagen, Denmark). Raw reads imported from IonTorrent were screened for chimeras, and reads <150 bp and with Q score <25 were removed. Good quality reads were clustered into OTUs (operational taxonomic units) at 97% sequence identity, and the OTUs were assigned taxonomically using a RDP classifier^22^ with reference databases RDP 16S rRNA training set 16 and UNITE Fungal ITS trainset 7.1^23^ for bacteria and fungi, respectively (https://rdp.cme.msu.edu). OTUs representing plant chloroplast or mitochondrial sequences, as well as OTUs with <10 reads were removed from the dataset prior to analyses.

Square root transformed data was used to construct Bray-Curtis distance matrixes, which were used to analyze community structures using PERMANOVA (permutational multifactorial manova)^24^ and visualized by PCoA ordinations at the OTU level. Taxonomic groups (phyla or OTU) with the strongest impact on differences between community structures were identified with SIMPER (Similarity Percentages - species contributions), all performed with PRIMER 6.1 software package with the PERMANOVA+ add-on (primer-e.com).

IonTorrent sequencing resulted in 270705 fungal and 322177 bacterial quality filtered sequence reads. After sequence data processing (removal of chimeric sequences, low read count samples, OTUs with less than 10 reads and plant mitochondrial sequences), fungal (ITS) and bacterial (16S rRNA) datasets consisted of 214304 and 167772 sequence reads assigned into 54 and 233 OTUs, respectively.

The ITS dataset was reanalyzed after removing the *Epichloë* assigned reads. After depletion, five E+ samples had less than 90 reads. Those samples were removed from the dataset, along with five randomly selected E− plant samples, and thereafter 10 samples from both E+ and E− plants were used for community analyses.

Raw sequence data are available at the EBI Sequence Read Archive under accession PRJEB29516.

## Results

### Fungal endophytic communities are strongly shaped by *Epichloë* symbiosis

**Figure 1:**
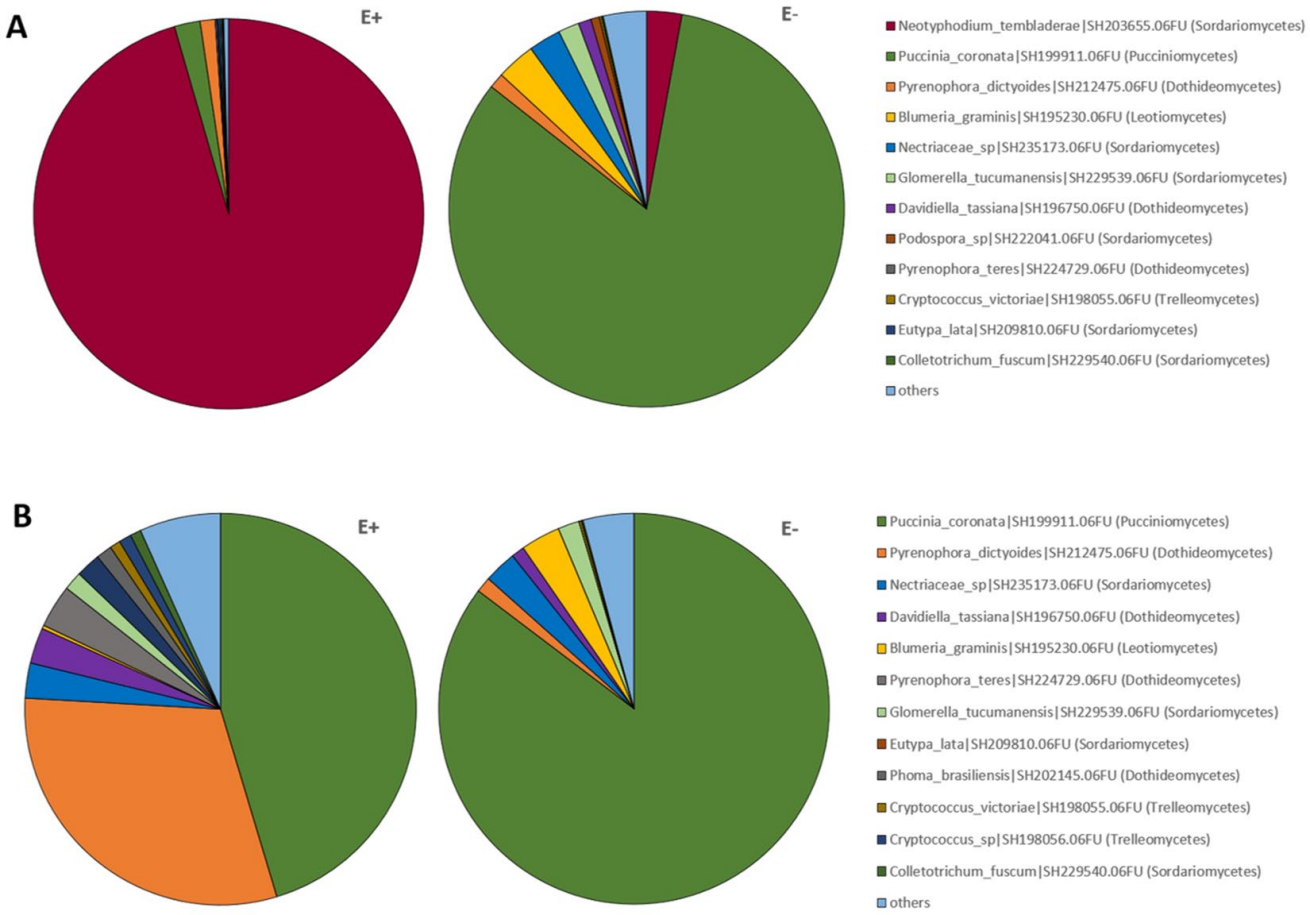
Taxonomic composition of *Schedonorus phoenix* endophytic fungal communites in *Epichloë* colonized (E+) and uncolonized (E−) plants. (**A**) total fungal communities, (**B**) fungal communities based on *Epichloë* depleted dataset. Fungal communities are presented at fungal genus level. 15 biological replicates of both E+ and E− plants were used in analysis of total communities, while 10 biological replicates of E+ and E− plants were used in Epichloë depleted dataset analysis. Neotyphodium = *Epichloë*.

**Figure 2:**
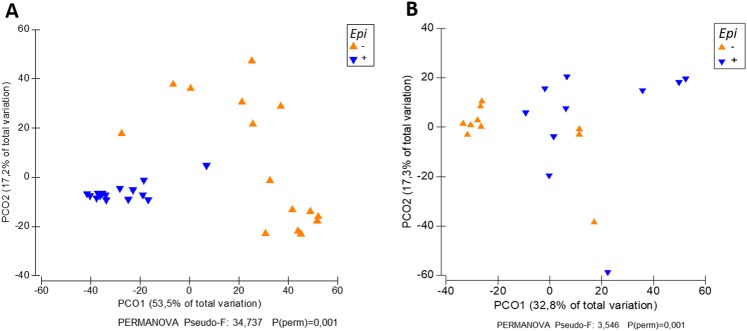
Principal coordinate analysis (PCoA) of endophytic fungal communities in E+ and E− plants. (**A**) total communities, (**B**) fungal communities based on *Epichloë* depleted dataset. PCoAs are based on Bray-Curtis distance matrices of standardized, square root transformed community data of 15 (**A**) and 10 (**B**) biological replicates of E+ and E− plants.

The fungal endophytic communities in tall fescue (*Schedonorus phoenix*) leaf samples represented 17 classes in the fungal phyla Ascomycota, Basidiomycota and Zygomycota. Communities in the plants colonized by *Epichloë* (E+ plants) were very strongly dominated by *Epichloë* (fungal class Sordariomycetes) (96% relative abundance of the communities in the E+ plants), followed by OTUs assigned as *Puccinia coronata* (class Pucciniomycetes) and *Pyrenophora dictyoides* (class Doditheomycetes) (2% and 1% relative abundance in E+ plant communities, respectively) (Fig. 1). E− plant communities were dominated by *Puccinia coronata* (Pucciniomycetes), with 83% relative abundance, followed by *Blumeria graminis* (Leotiomycetes, 3%) and Nectriaceae sp., *Epichloë* (both class Sordariomycetes, 6%), *Glomerella tucumanensis*, *Pyrenophora dictyoides* and *Davidiella tassiana*. Other OTUs were present at relative abundances of less than 1% (Fig. 1). Endophytic fungal communities in E+ and E–plants differed significantly in their community structures (PERMANOVA p = 0.001), which was also clearly visible in PCO ordination (Fig. 2A).

To gain a better insight in the endophytic fungal communities other than the systemic *Epichloë* symbiont, we reanalyzed the data after removing *Epichloë*-assigned OTU reads from the dataset. In the *Epichloë*-depleted datasets, *Puccinia coronata* was the most abundant OTU in both E+ and E− plant communities (45% and 85% of the total relative abundance, respectively), followed by *Pyrenophora dictyoides* and *P*. *teres* in E+ plants (31% and 4%, respectively), and Nectriaceae sp. and *Glomerella tucumanensis* in E− plants (2 and 1%, respectively) (Fig. 1B). PERMANOVA analysis of the *Epichloë* depleted communities demonstrated, that there was small but significant difference in the fungal endophyte community structures between E+ and E− plants (Fig. 2B). SIMPER (similarity percentages species contribution) analysis identified *Puccinia coronata*, *Pyrenophora dictyoides* and *Nectriaceae* sp. as the major taxa driving these differences (Table 1). Several other taxa, including OTUs representing *Phoma* sp. and *Davidiella* sp. were also differentially abundant in E+ and E− plants.

**Table 1:** SIMPER (Similarity percentage species contribution) analysis of fungal taxa contributing to the differences in community structures between E+ and E− plants, based on Bray-Curtis dissimilarity matrix of standardized community data.

Species	ref seq ID (Unite)	E−	E+	AvDi	Di/SD	Co%	Cu%
		AvAb	AvAb				
**A Groups E− & E+, Average dissimilarity = 86,67**							
Neotyphodium (Epichloë)	SH203655.06 FU	9,54	91,88	4 1,21	4,22	47,55	47,55
Puccinia_coronata	SH199911.06FU	52,71	4,40	25,43	1,27	29,34	76,89
Nectriaceae_sp	SH235173.06FU	8,66	0,38	4,34	0,61	5,01	81,90
Davidiella_tassiana	SH196750.06FU	6,66	0,58	3,40	0,51	3,92	85,82
Blumeria_graminis	SH195230.06FU	3,64	0,01	1,83	0,27	2,11	87,93
Glomerella_tucumanensis	SH229539.06FU	3,09	0,09	1,54	0,56	1,78	89,71
Pyrenophora_dictyoides	SH212475.06FU	1,58	1,62	1,37	0,57	1,58	91,29
**B Groups E− & E+** **Average dissimilarity = 70,13**							
Puccinia_coronata	SH199911.06FU	73,88	31,55	26,60	1,88	37,93	37,93
Pyrenophora_dictyoides	SH212475.06FU	0,72	20,99	10,45	0,72	14,91	52,84
Nectriaceae_sp	SH235173.06FU	6,04	14,30	7,82	1,18	11,15	63,99
Blumeria_graminis	SH195230.06FU	5,47	0,73	3,03	0,37	4,32	68,31
Phoma_brasiliensis	SH202145.06FU	0,00	5,67	2,83	0,48	4,04	72,35
Cryptococcus_sp	SH198056.06FU	0,01	4,40	2,20	0,44	3,14	75,49
Glomerella_tucumanensis	SH229539.06F	3,20	2,23	2,04	0,80	2,91	78,40
Chionosphaeraceae		0,00	3,88	1,94	0,33	2,76	81,16
Rhizoscyphus_ericae	SH207166.06FU	0,00	2,86	1,43	0,33	2,04	83,20
Podospora_sp	SH222041.06FU	2,66	0,00	1,33	0,33	1,89	85,09
Davidiella_tassiana	SH196750.06FU	2,05	0,76	1,24	0,52	1,76	86,86
Fungi_unidentified_6		0,00	2,47	1,24	0,33	1,76	88,62
Pyronemataceae_sp	SH227977.06FU	0,00	1,63	0,82	0,33	1,16	89,78
Candida_smithsonii	SH216776.06FU	0,00	1,56	0,78	0,42	1,11	90,90

A: total fungal endophytic communities, B: communities with Epichloë assigned reads removed. AvAb: Average abundance, AvDi: Average dissimilarity, Di: Dissimilarity, SD: standard deviation, Co%: contribution to the observed dissimilarity, % of total, Cu%: cumulative contribution, %. Only OTUs up to 90% cumulative contribution are listed.

### Bacterial community structures are unaffected by *Epichloë* symbiosis

**Figure 3:**
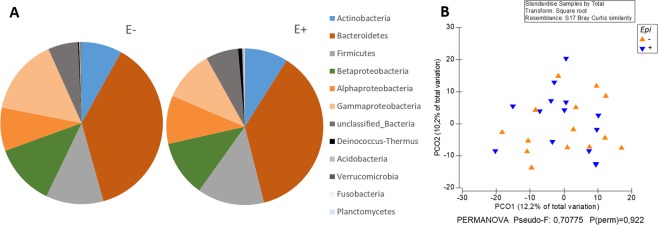
Taxomonic composition (**A**) and principal coordinate analysis (PCoA) of community structures (**B**) of endophytic bacterial communities in E+ and E− plants. PCoA is based on Bray-Curtis distance matrix of standardized, square root transformed community data.

Bacterial communities in *S*. *phoenix* plants were dominated by OTUs assigned to the phyla *Bacteroidetes* (five most abundant OTUs in the family *Chitinophagaceae*) and *Proteobacteria* (two OTUs in the family *Pseudomonadaceae*), which collectively comprised 37% and 34% of the total dataset reads, respectively. Community structures of the bacterial endophytic communities were not affected by *Epichloë* symbiosis (PERMANOVA p = 0.925, Fig. 3). Analysis was repeated at several levels of taxonomy (OTU at 99% identity, bacterial families, orders and classes), but no significant differences or trends were detected (data not shown).

## Discussion

In this study, we tested the hypothesis that systemic *Epichloë* endophytes as a keystone species would simultaneously shape plant fungal and bacterial communities in host plant endospheres. Our data partially supported the hypothesis. *Epichloë* symbiosis strongly shaped the endophytic fungal community of its host plant, *Schedonorus phoenix*. *Epichloë* dominated the fungal communities of the E+ plants and furthermore, relative abundances of other fungal taxa markedly differed from E− plants. When comparing fungal communities, excluding *Epichloë*, 45%, 31% and 4% of the total relative abundance were *Puccinia coronata*, *Pyrenophora dictyoides* and *P*. *teres* in E+ plants, respectively. In E− plants, latent infections of common pathogen *P*. *coronata* strongly dominated the fungal communities (85% of total relative abundance), followed by *Blumeria graminis* (3%), Nectriaceae sp. (2%) and *Glomerella tucumanensis* (1%). Several other taxa were also differentially abundant in E+ and E− plants. In general, these results suggest that fungal endophyte communities are dominated by one keystone species that shapes the taxonomic composition of rest of the fungal community. In the case of *Epichloë* species noteworthy is that the dominance is heritable in mother plant lineages. In contrast to our hypothesis, however, bacterial community structures in *S*. *phoenix* plants appeared to be unaffected by *Epichloë* presence.

Negligible effects of *Epichloë* on the bacterial community in *S*. *phoenix* plants suggests that *Epichloë* modulates the fungal community either via direct fungus-fungus interactions, such as competition (space occupation, nutrient limitation) or antagonism, or fungal-specific molecular or physiological mechanisms. Systemic *Epichloë* species have been shown to modulate plant physiology and metabolism on several fronts, including increased production of alkaloids and phenolic root exudates, and upregulation of systemic hormone and defense responses^12,25–27^. Plant associated bacterial communities have been reported to respond to changes in many or all of these aspects^28,29^. Thus, we expected to see an impact of *Epichloë* colonization status on bacterial community structures as well.

Contrasting findings have been reported on impact of *Epichloë* colonization on plants in past literature. While several studies report significant changes in plant gene regulation and systemic changes in synthesis and processing of the plant hormones salicylic acid, jasmonate, gibberellin, ethylene, abscisic acid, cytokinin and auxin^26,27,30^, others report only subtle differences in gene expression and a lack of detectable ultrastructural changes^31,32^. These contrasting plant responses to fungal endophytes are most likely explained by different combinations of symbiont and plant species with different symbiotic histories of host plants as well as by experimental conditions^6,8,13^. Studies reporting significant shifts in plant gene expression in response to *Epichloë* are mainly conducted with artificial fungus-plant combinations and using fungal removal and/or inoculation manipulations^26,27,30^, while studies reporting subtle plant responses have been conducted with E+ plant lines with long symbiotic histories and naturally endophyte-free E− plants^31^. Plants in our experiment were naturally colonized by compatible, vertically transmitted endophyte. Lack of plant transcriptional responses in naturally compatible *Epichloë*-plant symbiosis^33^ combined with our observations of highly similar bacterial communities in E+ and E− plants suggests that one of the major triggers of plant defense, recognition of non-self ^34^, is not induced in compatible *Epichloë* symbiosis of long symbiotic history.

In short, our results highlight the importance of comprehensive phytobiome-level studies on bacterial and fungal interactions. We demonstrated that *Epichloë* species can be regarded as keystone species in shaping fungal communities. However, the observed divergent impact of *Epichloë* on bacterial and fungal communities in the leaf endosphere of *S*. *phoenix* supports the idea of the context dependency of microbial interactions. Although the modulation of fungal communities by *Epichloë* species appears to be directed via fungal-fungal-interactions rather than via modulation of counteracting SA and JA regulated plant response pathways, we cannot rule out the pivotal role of signaling and chemical cross-talk among fungal and bacterial members of plant associated microbiomes. We may expect that horizontally transmitted micro-fungi with short evolutionary history with their hosts may affect host quality of the bacterial community as well. Future studies will reveal whether the remarkable subtlety in *Epichloë*-plant symbiosis has evolved to block/prevent some conserved induced molecular or physiological plant responses to other microbes.
